# Supplementary material for: A high-resolution mRNA expression time course of embryonic development in zebrafish
Source: eLife. 2017 Nov 16;6:e30860. doi: 10.7554/eLife.30860 (PMC5690287; doi:10.7554/eLife.30860)
Supplement: Supplementary file 6. [file elife-30860-supp6.zip › biolayout-clusters-files/Cluster241-genes.html]

Cluster241


# Cluster241: Genes

| Ensembl ID | Gene Name | Chr | Start | End | Biotype |
| --- | --- | --- | --- | --- | --- |
| ENSDARG00000077357 | LRRC61 | 25 | 25642308 | 25652391 | protein\_coding |
| ENSDARG00000058094 | ciarta | 19 | 9009509 | 9021143 | protein\_coding |
| ENSDARG00000038094 | clec16a | 3 | 26692917 | 26775785 | protein\_coding |
| ENSDARG00000061738 | march2 | 22 | 17484444 | 17496315 | protein\_coding |
| ENSDARG00000086405 | mterf2 | 18 | 15179419 | 15187450 | protein\_coding |
| ENSDARG00000039681 | samm50l | 4 | 9010909 | 9024183 | protein\_coding |
